# Post-radioiodine management of patients with Graves' disease

**DOI:** 10.1186/1753-6561-6-S4-O37

**Published:** 2012-07-09

**Authors:** K Collins, P Perros, J Horsefield

**Affiliations:** 1 Departments of Endocrinology and Medical Physics, Royal Victoria Infirmary, Newcastle upon Tyne, UK

## Introduction

Radioiodine is a safe and effective treatment for Graves' disease. Iatrogenic hypothyroidism is very common after treatment, but its onset is unpredictable. Even a short episode of hypothyroidism can result in significant morbidity and ideally should be avoided. In Newcastle a standard dose of radioiodine (400MBq) is used, but for historical reasons two different protocols are used after radioiodine: Regimen A: regular clinical and biochemical monitoring and initiation of levothyroxine when serum thyroid hormones have normalized, and Regimen B: block and replace with Carbimazole and levothyroxine starting 2 weeks post-radioiodine and continuing for 6 months, then withdrawing Carbimazole, but continuing with levothyroxine long-term.

## Methods

The objective was to compare the two protocols for incidence of biochemical and clinical hypothyroidism during a 12 month post-radioiodine follow-up period and effects on weight gain and development or progression of orbitopathy. Patients with Graves' disease who were treated between January 2008-December 2009 were included. The medical records were reviewed and data were collected and analyzed.

## Results

One hundred and twenty two patients were studied, 78 treated with Regimen A and 43 with Regimen B. Euthyroidism at 8 weeks, 6 months and 12 months post-radioiodine was achieved in 50%, 64% and 73% of patients with Regimen A and 65.1%, 71% and 65% in patients with regimen B respectively. Clinical hypothyroidism during follow-up was commoner in Regimen A than B (52.6% vs 16.3% respectively, p<0.05). Weight gain was reported more frequently in Regimen A than B (43.6% vs 20.9%, p<0.05). The incidence of new Graves' orbitopathy developing after radioiodine was higher in Regimen A than B (11.1% vs 5.3%).

## Conclusions

A 6 month course of block and replace followed by levothyroxine after a standard 400MBq dose of radioiodine is associated with better clinical outcomes than a watchful approach and initiation of levothyroxine based on biochemical and clinical indicators.

